# Metabolism gene expression in worker honey bees after exposure to 50Hz electric field - semi-field analysis

**DOI:** 10.1186/s12983-024-00535-1

**Published:** 2024-05-29

**Authors:** Agnieszka Murawska, Paweł Migdał, Moritz Mating, Paweł Bieńkowski, Ewelina Berbeć, Ralf Einspanier

**Affiliations:** 1 Department of Bees Breeding, Institute of Animal Husbandry, Wroclaw University of Environmental and Life Sciences, Wroclaw, 51-630 Poland; 2 Institute of Veterinary Biochemistry, Freie Universitaet Berlin, Berlin, 14163 Germany; 3 Telecommunications and Teleinformatics Department, Wroclaw University of Science and Technology, 27 Wybrzeże Wyspiańskiego St., Wroclaw, 50-370 Poland

**Keywords:** Gene expression, Long-term effects, E-field exposure, 50Hz

## Abstract

The investigation of the effects of artificial 50 Hz electric field (E-field) frequency on *Apis mellifera* is a relatively new field of research. Since the current literature focuses mainly on short-term effects, it is unknown whether E-fields have permanent effects on bees or whether their effects can be neutralized. In this study we assessed gene expression immediately after exposure to the E-field, as well as 7 days after exposure. The aim of this work was to identify potentially dysregulated gene transcripts in honey bees that correlate with exposure time and duration to E-fields.

Newly emerged bees were marked daily with a permanent marker (one color for each group). Then bees were exposed to the 50 Hz E-field with an intensity of 5.0 kV/m or 10.0 kV/m for 1–3 h. After exposure, half of the bees were analyzed for gene expression changes. The other half were transferred to a colony kept in a mini-hive. After 7 days, marked bees were collected from the mini-hive for further analysis. Six regulated transcripts were selected of transcripts involved in oxidative phosphorylation (COX5a) and transcripts involved in endocrine functions (HBG-3, ILP-1), mitochondrial inner membrane transport (TIM10), and aging (mRPL18, mRPS30).

Our study showed that in *Apis mellifera* the expression of selected genes is altered in different ways after exposure to 50 Hz electric fields -. Most of those expression changes in Cox5a, mRPL18, mRPS30, and HGB3, were measurable 7 days after a 1–3 h exposure. These results indicate that some E-field effects may be long-term effects on honey bees due to E-field exposure, and they can be observed 7 days after exposure.

**Supplementary Information:**

The online version contains supplementary material available at 10.1186/s12983-024-00535-1.

## Background

While pollinators including the honey bee (*Apis mellifera* L.) search and collect food and water, they are exposed to many biological (pathogens, parasites, predators), chemical (e.g. substances used in agriculture), and physical stressors (e.g. electromagnetic) [1–4]. The electromagnetic fields in the bee environment cover a variety of frequencies - from low (1–50 Hz) to high (up to 5 GHz - wireless LAN) [5]. Bees may not only be exposed to such electromagnetic fields from various artificial sources in urban areas, but also in rural areas e.g. power lines, transmission towers, base transceiver stations, Wi-Fi networks, electrical devices and installations [6]. Honey bees flying at a height of about 2 m near a power line are exposed to electric field (E-field) of 50 Hz with an intensity of 10 to 12 kV/m. Approximately 5 m or more above the ground, a honey bee is exposed to an E-field of 5–7 kV/m. This occurs when high obstacles stand in its way [7, 8]. The investigation of the effects of artificial 50 Hz E-field frequency on *Apis mellifera* is a relatively new field of research. Recently, scientists demonstrated that 50 Hz E-field- with intensity levels of 5.0, 11.5, 23.0, and 34.5 kV/m and different exposure times change honey bee activity and biochemical parameters under laboratory conditions [3, 9–13]. A 50 Hz frequency is used in most countries for power systems [14]. The E-field-exposed bees showed less self-grooming and contact with other individuals than the control bees [3, 10]. Other studies found that bees’ abilities to learn, orientation, and feed or walk were reduced after acute exposure to a 50 Hz low-frequency E-field [15]. 60 Hz E-field with an intensity above 150 kV/m caused vibrations in the honey bee wings, antennae and hair. Their behaviour, shown by the “hot foot” reaction and stinging activities, was altered only if the bee was on a conductive substrate [16]. Furthermore, because of exposure to 50 Hz E-fields with the intensity of 5.0 kV/m, 11.5 kV/m, 23.0 kV/m, and 34.5 kV/m for 1, 3, 6, and 12 h, the activity of antioxidant enzymes (superoxide dismutase (SOD), catalases (CAT), and acidic, neutral, and alkaline proteases were altered [3, 11–13]. Additionally, Migdał et al. [15] found that changes in the concentration of total protein, glucose, and triglycerides in worker bee haemolymph are linked to the intensity and exposure time of the treatment. This indicates a relationship between the level of nutrients in bee haemolymph and the action of E-fields with a frequency of 50 Hz and various intensities. Such changes can lead to disturbances in energy production in the mitochondria and disorders in the metabolism of proteins, fats, and sugars [17]. The study presented here aimed to investigate the effects of honey bees exposure to 50 Hz E-fields with. For this purpose, one-day-old worker bees were exposed to an E-field and checked after exposure and seven days later. The collected material was analysed through expression analysis of transcripts involved in oxidative phosphorylation (COX5a) and transcripts involved in endocrine functions (HBG-3, ILP-1), mitochondrial inner membrane transport (TIM10), and aging (mRPL18, mRPS30), by qPCR. Transcripts were chosen because they regulate crucial biological processes like energy metabolism, aging, and nurse bee conversion to foragers. There has already been evidence that these transcripts are affected by other stressors [18–21]. Oxidative phosphorylation plays an important role in energy metabolism and consists of various complexes such as NADH dehydrogenase. The transcript cox5a, studied here encodes an important protein of the cytochrome C oxidase complex [22]. Therefore, altered expression of these transcripts could be the molecular reason for changes in mitochondrial metabolism and reduced lifetime. The endocrine transcripts analysed in the present study play an important role in the transition of nurse bees to foragers and expression levels differ between nurse bees and foragers [23]. Nurses and foragers differ in mRNA levels of specific genes including buffy, mRPS and others (vitellogenin, hbg3, mmp1, Kr-h1), and this is associated with differences in behaviour [24]. It can be dangerous for the entire honey bee colony if these processes are disrupted.

## Materials and methods

### Rearing worker honey bees

**Fig. 1:**
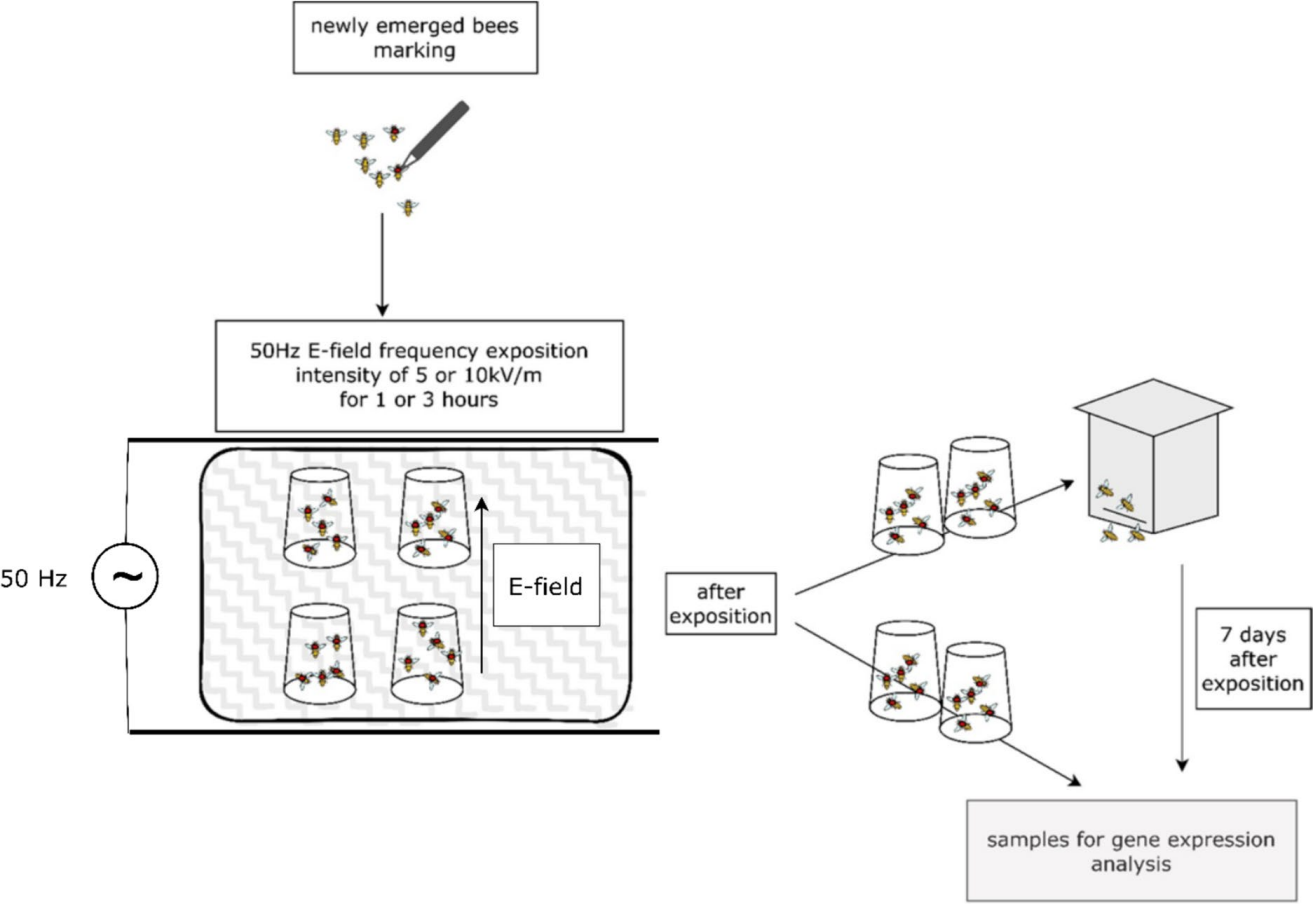
Experimental design: one-day old worker bees were exposed to the 50 Hz E-field with the intensity of 5.0 or 10.0 kV/m for 1–3 h

Frames with capped brood were taken from colonies with queens originated from the same mother-queen *Apis mellifera carnica* from the apiary of the Institute of Veterinary Biochemistry, Freie Universitaet Berlin, Berlin, Germany (52.42898 °N, 13.23762 °E*)* and put into an incubator with temperature of 35.5 °C ± 0.5 °C and relative humidity of 70% ± 5%) to emerge. Honey and bee pollen were provided *ad libitum* until the bees were transferred to the cages. Newly emerged bees were marked daily with a permanent marker (one colour for each group). Each group consisted of 300 bees. After exposure, 15 bees were analysed for gene expression changes (5 replications with 3 bees each). The half (150 bees) were transferred to a colony kept in a mini-hive. After 7 days, marked bees were collected from the mini-hive for gene expression analysis (Fig. 1). After 7 days were analysed gene expression in 15 bees (5 replications with 3 bees each). The mini-hive was located outside and bees could fly freely, allowing the colony to behave naturally. However, because of their age (~ 7 days), experimental bees didn’t leave the hive for long time.

### E-field set up

One-day old worker bees were put into plastic cages (dimension of ø_1_ = 55 mm, ø_2_ = 95 mm, h = 130 mm). In the experimental groups one-day old worker bees were exposed to the 50 Hz E-field (ELF -Extremely Low Frequency) with the intensity of 5.0 or 10.0 kV/m for 1–3 h. The measured value of E-field in the area where mini-hive were kept was < 1.0 kV/m. Bees from control group were not exposed to the E-field but marked with a marker and placed in the same hive as the experimental group. In the exposure system, a homogeneous 50 Hz E-field was generated using a plate capacitor as described by Migdał et al. [11]. The field intensity was fixed to 5.0 kV/m and 10.0 kV/m.

The heterogeneity and fluctuations of field intensity in the exposure area did not exceed 5%. The field intensity and its distribution were measured by an Electromagnetic Field Standards and Metrology Laboratory accredited testing laboratory PCA AB-361using the ESM-100-meter No. 972,153 with the calibration certificate LWiMP/W/056/2021 of February 15, 2023, issued by the accredited calibration laboratory PCA AP-078. The stability of the field strength during the experiment was monitored by controlling the voltage applied to the exposure system.

### Selected gene expression analysis

Worker honey bees were exposed to the E-field for 1–3 h. 1 h after exposure, the bees were frozen for analysis, the next part of bees was frozen 7 days after exposure (15 worker bees were taken each frozen), frozen in liquid nitrogen and then stored at -80 °C until further use. Six significantly regulated transcripts were selected related to energy production and metabolism in mitochondria: Cox5a, mRPL18, mRPS30, ILP1, HBG3, and M10. For all genes the reference genes were RPS5 and RPS18 [25]. The primers used for gene expression analysis are listed in Table 1.

**Table 1:** Primers used in gene expression analysis

Target gene	Primers	Sequence for & rev (5’-3’)	GenBank No.
Cox5a	ame COX5a for	TCGCATGATGGACCACAAGA	XM_392368.6
	ame COX5a rev	AGGTACAAGATCCATAGCCGC	
mRPL18	ame mRpL18 for	ACTGCATTTTGGCATAAACTTGA	XM_625002.5
	ame mRpL18 rev	TGCAAGCACACGTCCTACAA	
mRPS30	ame mRpS30 for	ACAGGCTTGTTATCAAGGTTTTTCA	XM_396435.6
	ame mRpS30 rev	TGACCAGTATTGGCCATTTGTT	
ILP1	ame ILP1 for	GGGGTACCATGGGAAGTAACCGTCCTAAG	AB253763.1
	ame ILP1 rev	ACGCGTCGACTCAAACTGCCTCCTTAAGATT	
TIM10	ame Tim10 for	TCGCATAATAGCGTGGTCACA	XM_006571277.2
	ame Tim10 rev	ACCATCCAATCGCTATCTTCGT	
HBG3	ame HBG3 for	TACCTGGCTTCGTGTCAAC	NM_001011608.1
	ame HBG3 rev	ATCTTCGGTTTCCCTAGAGAATG	

### RNA-extraction

RNA-extraction was performed using the Quick-RNA™ Miniprep Kit (Zymo Research Europe GmbH, Freiburg, DE). Briefly, 2 Individuals were pooled and lysed in a lysing Matrix S (MP Biomedicals, Heidelberg, DE) containing 800 µl of lysis buffer using a BeadBlaster (Benchmark Scientific, Edison, USA). Tubes were then centrifuged at 12.000 x g at 4 °C for 10 min. Supernatant was transferred into a clean microcentrifuge tube containing 1x volume 100% Ethanol. The solution was then used according to manufacturer’s protocol. RNA was eluted in a total volume of 50 µl ddH_2_O. Quantity and quality of total RNA was analysed using an Agilent RNA 6000 nano chip on a 2100 Bioanalyzer (Agilent Technologies, California, USA). Isolated RNA was stored at -80 °C until further use.

### First strand cDNA-Synthesis

Protoscript ® II Transcriptase (New England Biolabs, Inc., Ipswich, USA) has been used according to manufacturer’s protocol. Briefly, 1 µg DNA-free RNA has been incubated with 1 µl d(T)23VN-Primer (5µM) and 1 µl Random Primer Mix (5µM) at 65 °C for 5 min in a total volume of 8 µl. Thereafter 12 µl of Protoscript Mastermix was added and sample has been incubated at 42 °C for 60 min and heat inactivated at 80 °C for 5 min. cDNA was then diluted by addition of 80 µl ddH2O and stored at -20 °C in adequate aliquots.

### RT-qPCR

Expression analysis was performed by means of SYBR Green detection chemistry using the Biozym Blue S’Green 2× Mix and the PikoReal Real-Time PCR-System (Thermo Scientific). All reactions were carried out using clear PikoReal-96-well plate (Thermo Scientific) that were sealed with adhesive films. The following RT-qPCR protocol was applied: denaturation at 95 °C for 5 min, followed by 40 amplification cycles including 95 °C for 5 s and 60 °C for 30 s, the fluorescence signal was acquired at 60 °C. A subsequent melting curve (60–95 °C) was performed as quality control with continuous fluorescence measurement and final cooling to room temperature following a published protocol [26]. PCR reactions were performed using a master mix containing 2× Mix SYBR with 1 µl of diluted cDNA template. After dispensing 9 µl of master mix in respective sample wells of a 96 well plate, 0.5 µl of each forward and reverse primer (each 4 µM) were added and amplified as mentioned above. Samples containing water instead of RT-reaction served as negative controls.

### Statistical analysis

The normality of the data distribution was analysed using the Shapiro-Wilk test. The statistical significance of differences between groups was determined by the Kruskal-Wallis test using the package “pgirmess” for “kruscalmc” function with Holm correction. For all tests, RStudio was used with a significance level of α = 0.05.

## Results

**Fig. 2:**
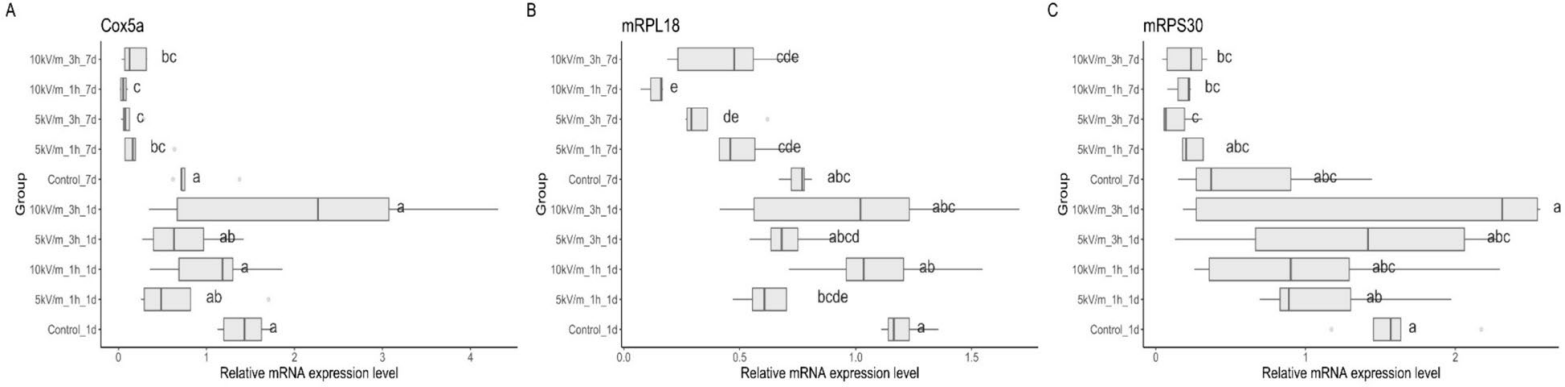
Cox5a (**A**), mRPL18 (**B**), mRPS30 (**C**) relative level of expression in the haemolymph of one-day and seven-day-old bees exposed to 50 Hz E-field with intensity of 5.0 or 10.0 kV/m for 1–3 h. The honey bee groups were named as follows: control group 1 day or 7 days = control1d, control7d; 5 kV/m treatment for 1 h either 1 or 7 day = 5 kV/m1h1d, 5 kV/m11h7d; 5 kV/m treatment for 3 h either 1 or 7 day = 5 kV/m3h1d, 5 kV/m3h7d; 10 kV/m treatment for 1 h either 1 or 7 day = 10 kV/m1h1d, 10 kV/m1h7d; 10 kV/m treatment for 3 h either 1 or 7 day = 10 kV/m3h1d, 10 kV/m3h7d. There were 5 replications with 3 bees for each group

One-day-old bees generally had higher Cox5a expression than seven-day-old bees, but not all differences were significant (Fig. 2A). Cox5a expression in one-day-old bees in treated groups was not different from the control group. However, in the case of seven-day-old bees, expression was significantly different between experimental and control groups. Comparing bees exposed for the same time to the same intensity, significant differences can be found after exposure to 10 kV/m for 1 and 3 h, and to 5 kV/m for 3 h between one-day-old and seven-day-old bees. There was no significant difference between one-day-old and seven-day-old control bees.

In most cases, seven-day-old bees in experimental groups had lower mRPL18 expression than control bees and one-day-old bees (Fig. 2B). mRPL18 expression in one day old bees in experimental groups was not different from the control group, except in the 5 kV/m1h1d group. In the case of seven-day-old bees, expression was significantly different between the 10kV1h7d group and control group, and the 5 kV/m3h7d group and control group. Comparing bees exposed for the same time to the same intensity, significant differences can be noticed between one-day-old and seven-day-old bees exposed to 10 kV/m for 1 h. There was no significant difference between one-day-old and seven-day-old control bees.

Seven-day-old bees in experimental groups had lower mRPS30 expression than control bees and one-day-old bees, but not all differences were significant (Fig. 2C). mRPS30 expression in bees in experimental groups was not different from the control group. Comparing bees exposed for the same time to the same intensity, no significant differences can be noticed between one-day-old and seven-day-old. There was no significant difference between one-day-old and seven-day-old control bees.

**Fig. 3:**
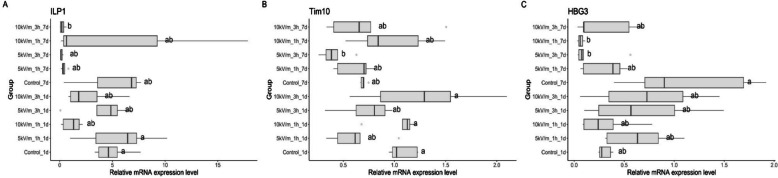
ILP1 (**A**), Tim10 (**B**) and HBG3 (**C**) relative level of expression in the haemolymph of one-day and seven-day-old bees exposed to 50 Hz E-field with intensity of 5.0 or 10.0 kV/m for 1–3 h. The honey bee groups were named as follows: control group 1 day or 7 days = control1d, control7d; 5 kV/m treatment for 1 h either 1 or 7 day = 5 kV/m1h1d, 5 kV/m11h7d; 5 kV/m treatment for 3 h either 1 or 7 day = 5 kV/m3h1d, 5 kV/m3h7d; 10 kV/m treatment for 1 h either 1 or 7 day = 10 kV/m1h1d, 10 kV/m1h7d; 10 kV/m treatment for 3 h either 1 or 7 day = 10 kV/m3h1d, 10 kV/m3h7d. For each group was 5 replications with 3 bees

One-day-old bees had higher ILP1 and Tim10 expression than seven-day-old bees, but those differences were not significant (Fig. 3A, B). ILP1 and Tim10 expression in experimental groups was not different from that of the control group. One-day-old and seven-day-old bees exposed to the same intensity for the same time showed no significant differences. Gene expression in one-day-old control bees did not differ significantly from that of seven-day-old control bees.

HBG3 expression in one day old bees in experimental groups was not different from the control group (Fig. 3C). However, seven-day-old bees exposed to 10 kV/m for 1 h and to 5 kV/m for 3 h displayed significantly different expression levels than control group. Comparing bees exposed for the same time to the same intensity, no significant differences can be noticed between one-day-old and seven-day-old bees. There was also no significant difference between one-day-old and seven-day-old control bees.

## Discussion

Changes in honey bee gene expression detected in our experiment suggest alterations in mitochondrial metabolism and energy production. They may be related to the research of Migdal et al. in which it the formerly observed levels of total protein, glucose, and triglycerides in honeybee haemolymph. They showed changes the lowest protein concentration in worker honeybee haemolymph was found in the control group (0.13 mg/mL), but the highest protein concentrations in the experimental groups were exposed to 23.0 kV/m and 34.5 kV/m for 12 h. Furthermore, glucose concentrations in bee haemolymph were lower in experimental groups. The E-field also decreased triglycerides concentration in bee haemolymph [9]. In our previous study, we showed that immediately after exposure to 50 Hz E-fields with the intensity of 5.0 kV/m, 11.5 kV/m, 23.0 kV/m, and 34.5 kV/m for 1, 3, 6, or 12 h, the activity of antioxidant enzymes (such as superoxide dismutase (SOD) and catalase (CAT)) and nonenzymatic antioxidant concentration (creatinine and albumin) in workers’ haemolymph changed [11, 12]. Furthermore, AST, ALT, and ALP activity in honey bees’ haemolymph significantly decreased [12], and neutral, and alkaline proteases activity was altered [3]. In this study, we measured gene expression one hour after exposure and 7 days later. In general, we found no significant expression changes of selected genes shortly after exposure in one-day-old bees in the experimental groups compared to the control group. While after 7 days, Cox5a, mRPL18 and HBG3 expression in the experimental groups differed from control bees, in many cases significantly lowered concentrations (Fig. 2). The result regarding changes 7 days after exposure is important because it indicates that the effect is observed only after a longer period and may cause long-lasting effects. Many changes occur during the life of honey bee workers, the first visible changes occur on the 7th − 8th day after hatching, the mushroom body in the brain grows, the microbiome stabilizes, and the diet and vitellogenin level change. All this also affects the expression of selected groups of genes [27–32]. If changes occur during this period caused by an external factor, it will affect the remaining periods of the worker’s life. This relationship was demonstrated in the work Goblirsch et al., where one-day-old bees were exposed to nosemosis, which resulted in a non-standard vitellogenin expression system and juvenile hormone levels [33]. In the study of Suzhen et al. for superoxide dismutase (SOD) after 7 days of exposure to flumethrin, its activity significantly decreased. Which resulted in changes in the body’s antioxidant level [34]. Bee colonies exposed to stress for a long time are characterized by a higher level of expression of aggression genes in the brain [35]. This is also confirmed by research by Christen et al. on the impact of short-term exposure of honey bee workers to neonicotinoids. They showed a significant increase of AChRα1, AChRα2, vitellogenin, and catalase transcripts after 24 h of exposure and persisted after cessation of exposure [36]. Our test results indicate no significant changes immediately after exposure. However, these changes are visible 7 days after exposure.

E-fields at 50 Hz alter honey bee behaviour, protease activity, antioxidant, and detoxification enzymes. Those changes may impair crucial metabolic cycles in honey bees [3, 9–13, 37].

To determine whether exposing honey bees to E-field can cause persistent changes and trigger a response in honey bee organism we analysed the expression profile of Cox5, mRPL18, mRPS30, ILP1, Tim10, and HBG3 genes.

Cytochrome C oxidase (COX) is a mitochondrial enzyme that functions at the end of the respiratory chain and plays a key role in the regulation of aerobic production of energy [38, 39]. It is not yet fully understood what its subunits do, but it is known that they play a role in enzyme assembly/stability, and dimerization. Furthermore, they protect the catalytic core from reactive oxygen species (ROS) and modulate the enzyme’s catalytic activity [39–41]. Cox5a is involved in oxidative phosphorylation. In our study, seven-day-old bees in all experimental groups had significantly lower Cox5a expression than control bees (Fig. 2A). Altered expression of Cox5a can reduce lifespan or change flight behaviour. The shortened lifespan of individuals may weaken the bee colony, just as a disturbed flight may cause problems collecting nectar and returning to the hive [21].

In model organisms (such as mice and nematode *Caenorhabditis elegans*) mitochondrial ribosomal protein (MRP) genes have been linked to aging and longevity [42, 43]. The mRPL18 gene encodes a protein component of the larger 39 S subunit of the mitochondrial ribosome. It may also assist in mitochondrial import of nucleic-encoded 5 S rRNA [44]. mRPS30 is a protein coding gene. Its related pathways include mitochondrial translation and protein metabolism [45]. In this study, seven-day-old bees in experimental groups (10 kV/m1h and 5 kV/m3h7d) had significantly lower mRPL18 expression than control bees (Fig. 2B). This may reduce the production of energy necessary for protein metabolism and translation. All this can translate into cellular metabolism and its functioning.

HBG3 encode alpha-glucosidase, enzyme which converts nectar sucrose into glucose and fructose [46, 47]. This enzyme occurs in the hypopharyngeal glands of worker honeybees - nurse bees secrete mainly major royal jelly proteins, whereas foragers secrete mainly alpha-glucosidase III [48]. In this study, we noticed significantly lower HBG3 expression in seven-day-old bees in 10 kV/m1h7d and 5 kV/m3h7d groups than in control bees (Fig. 3C). HBG3, among other genes, is involved in the transition of nurse to forager bees [24]. The above change may disturb the biological composition of the bee colony by reducing the number of foragers.

Many scientific studies focus on the impact of electromagnetic fields (EMF) in different frequencies on animals, including humans, and its possible impact on health [49–51]. Publications in the last two decades show the impact of radio waves (900 and 902 MHz) not only on individual cells, their genetic information (DNA) or tissues, but also on learning, concentration, problem solving and human cognitive skills [52, 53]. In the case of nematodes (*Caenorhabditis elegans*) waves 50 MHz to 1 GHz with prolonged exposure resulted in thermal shock, accelerated puberty by 40% and increased stress hormone concentration compared to the control group [54]. Studies indicate EMF (50 Hz, 1.05 mT) cytogenetic effect on lymphocytes [55]. In this context it was reported that extremely low frequency E-fields can contribute to changes in methylation in the mouse genome (50 Hz 1 mT, 2 mT, and 3 mT), learning abilities (50 Hz 1mT, 8mT) and body weight (50 Hz 250 µT, 500 µT, or 1 mT) [56–59]. The grapevine snail (*Helix pomatia*) as a model organism was subjected to an E-field of various frequencies from 8 Hz to 300 Hz. In the case of temporary exposure, hyperpolarization of nerve cells was observed [60]. Exposure from 0.5 to 120 h caused mainly a linear increase in mortality, while long-term exposure (2 months) caused changes at the cellular level contributing to DNA integrity loss, damage to lysosome membranes and oxidation disorders [61].

## Conclusion

Our study showed that the expression of selected genes in the honey bee *Apis mellifera* is altered in different ways after exposure to electric fields (E-field). Most of those expression changes, which are mainly related to mitochondrial metabolism and energy production, were measurable only 7 days after a 1- or 3 h exposure. These results indicate that some EMF effects are not be observed immediately after exposure, but that there may be long-term effects on honey bees. Finally, these molecular changes in transcript pattern detected here for the first time need to be investigated further in order to elucidate their effects both on the individual bee and on the entire colony.

### Supplementary Information

Supplementary Material 1.

## Data Availability

The datasets generated during and/or analysed during the current study are available from the corresponding author on reasonable request.

## References

[1] Genersch E (2010). Honey bee pathology: current threats to honey bees and beekeeping. Appl Microbiol Biotechnol.

[2] De la Rúa P, Jaffé R, Dall’Olio R, Muñoz I, Serrano J (2009). Biodiversity, conservation and current threats to European honeybees. Apidologie.

[3] Migdał P, Murawska A, Strachecka A, Bieńkowski P, Roman A. Honey bee proteolytic system and behavior parameters under the influence of an electric field at 50 hz and variable intensities for a long exposure time. Animals. 2021;11:863. 10.3390/ani11030863 PMC8003097 33803600

[4] Vanbergen AJ (2013). Threats to an ecosystem service: pressures on pollinators. Front Ecol Environ.

[5] Kim S, Kim K, Lee JH, Han SH, Lee SH. Differential expression of acetylcholinesterase 1 in response to various stress factors in honey bee workers. Sci Rep. 2019;9:10342. 10.1038/s41598-019-46842-0 PMC6637154 31316163

[6] Ioriatti L, Martinelli  M, Viani F, Benedetti M, Massa A (2009). Real-time distributed monitoring of electromagnetic pollution in urban environments. International Geoscience and Remote Sensing Symposium (IGARSS).

[7] Marino AA, Becker R. High voltage lines: hazard at a distance. Environment. 1978;20(9);1–52.

[8] Djalel D (2014). Study of the Influence High-Voltage Power Lines on Environment and Human Health (Case Study: the Electromagnetic Pollution in Tebessa City, Algeria). J Electr Electron Eng..

[9] Migdał P, Murawska A, Bieńkowski P, Strachecka A, Roman A. Effect of E-field at frequency 50 hz on protein, glucose, and triglycerides concentration in honeybee hemolymph. Eur Zoological J. 2021;88:1170–6.

[10] Migdał P, Murawska A, Bieńkowski P, Berbeć E, Roman A. Changes in honeybee behavior parameters under the influence of the e-field at 50 hz and variable intensity. Animals. 2021;11:247. 10.3390/ani11020247 PMC7909437 33498413

[11] Migdał P, Roman A, Strachecka A, Murawska A, Bieńkowski P (2020). Changes of selected biochemical parameters of the honeybee under the influence of an electric field at 50 hz and variable intensities. Apidologie..

[12] Migdał P, Murawska A, Bieńkowski P, Strachecka A, Roman A (2021). Effect of the electric field at 50 hz and variable intensities on biochemical markers in the honey bee’s hemolymph. PLoS ONE..

[13] Migdał P, Murawska A, Strachecka A, Bieńkowski P, Roman A (2020). Changes in the Honeybee antioxidant system after 12 h of exposure to Electromagnetic Field frequency of 50 hz and variable intensity. Insects..

[14] Götz M, Rapp M, Dostert K. Power line channel characteristics and their effect on communication system design. IEEE Commun Mag. 2004;42:78–86.

[15] Shepherd S, Lima MAP, Oliveira EE, Sharkh SM, Jackson CW, Newland PL. Extremely low frequency Electromagnetic fields impair the cognitive and motor abilities of Honey bees. Sci Rep. 2018;8:7932. 10.1038/s41598-018-26185-y PMC5962564 29785039

[16] Bindokas VP, Gauger JR, Greenberg B. Laboratory investigations of the electrical characteristics of honey bees and their exposure to intense electric fields. Bioelectromagnetics. 1989;10:1–12. 10.1002/bem.2250100102 2712835

[17] Migdał P, Murawska A, Roman A (2020). A modified standardized method to Extract and Store Insect Hemolymph with Use of a Glass Capillary. J Apic Sci.

[18] Christen V, Grossar D, Charrière JD, Eyer M, Jeker L. Correlation between increased homing flight duration and altered gene expression in the brain of Honey Bee foragers after acute oral exposure to Thiacloprid and Thiamethoxam. Front Insect Sci. 2021;1:765570. 10.3389/finsc.2021.765570 PMC10926505 38468880

[19] Christen V, Krebs J, Fent K. Fungicides chlorothanolin, azoxystrobin and folpet induce transcriptional alterations in genes encoding enzymes involved in oxidative phosphorylation and metabolism in honey bees (Apis mellifera) at sublethal concentrations. J Hazard Mater. 2019;377:215–26. 10.1016/j.jhazmat.2019.05.056 31170570

[20] Christen V, Krebs J, Bünter I, Fent K. Biopesticide Spinosad induces transcriptional alterations in genes associated with energy production in honey bees (Apis mellifera) at sublethal concentrations. J Hazard Mater. 2019;378:120736. 10.1016/j.jhazmat.2019.06.013 31202068

[21] Christen V. Different effects of pesticides on transcripts of the endocrine regulation and energy metabolism in honeybee foragers from different colonies. Sci Rep. 2023;13:1985. 10.1038/s41598-023-29257-w PMC9898565 36737645

[22] Mao W, Schuler MA, Berenbaum MR. Disruption of quercetin metabolism by fungicide affects energy production in honey bees (Apis mellifera). Proc Natl Acad Sci U S A. 2017;114:2538–43 . 10.1073/pnas.1614864114 PMC5347564 28193870

[23] Christen V, Kunz PY, Fent K. Endocrine disruption and chronic effects of plant protection products in bees: can we better protect our pollinators? Environ Pollut. 2018;243:1588–1601. 10.1016/j.envpol.2018.09.117 30296754

[24] Fent K, Haltiner T, Kunz P, Christen V. Insecticides cause transcriptional alterations of endocrine related genes in the brain of honey bee foragers. Chemosphere. 2020;260:127542 10.1016/j.chemosphere.2020.127542 32683019

[25] Jeon JH, Moon KH, Kim YH, Kim YH. Reference gene selection for qRT-PCR analysis of season- and tissue-specific gene expression profiles in the honey bee Apis mellifera. Sci Rep. 2020;10:13935. 10.1038/s41598-020-70965-4 PMC7435199 32811887

[26] Scholven J, Taras D, Sharbati S, Schön J, Gabler C, Huber O et al. Intestinal expression of TFF and related genes during postnatal development in a piglet probiotic trial. Cell Physiol Biochem. 2009;23:143–56. 10.1159/000204103 19255509

[27] Fahrbach SE, Moore D, Capaldi EA, Farris SM, Robinson GE. Experience-expectant plasticity in the mushroom bodies of the honeybee. Learn Memory. 1998;5:115–23. PMC311234 10454376

[28] Fluri P, Lüscher M, Wille H, Gerig L. Changes in weight of the pharyngeal gland and haemolymph titres of juvenile hormone, protein and vitellogenin in worker honey bees. J Insect Physiol. 1982;28:61–8.

[29] Haydak M. Bee Nutrition and Pollen substitutes. Apiacta. 1967;1:3–8.

[30] Ismail N, Robinson GE, Fahrbach SE. Stimulation of muscarinic receptors mimics experience-dependent plasticity in the honey bee brain. Proc Natl Acad Sci U S A. 2006;103:207–11. 10.1073/pnas.0508318102 PMC1324993 16373504

[31] Farris SM, Robinson GE, Fahrbach SE. Experience- and age-related outgrowth of intrinsic neurons in the mushroom bodies of the adult worker honeybee. J Neurosci. 2001;21:6395–404. 10.1523/JNEUROSCI.21-16-06395.2001 PMC6763189 11487663

[32] Withers GS, Fahrbach SE, Robinson GE. Selective neuroanatomical plasticity and division of labour in the honeybee. Nature. 1993;364:238–40. 10.1038/364238a0 8321320

[33] Goblirsch M, Huang ZY, Spivak M. Physiological and behavioral changes in Honey bees (Apis mellifera) Induced by Nosema ceranae infection. PLoS ONE. 2013;8:e58165. 10.1371/journal.pone.0058165 PMC3590174 23483987

[34] Qi S, Niu X, Wang Dhui, Wang C, Zhu L, Xue X et al. Flumethrin at sublethal concentrations induces stresses in adult honey bees (Apis mellifera L). Sci Total Environ. 2020;700:134500. 10.1016/j.scitotenv.2019.134500 31627045

[35] Rittschof CC, Robinson GE. Manipulation of colony environment modulates honey bee aggression and brain gene expression. Genes Brain Behav. 2013;12:802–11. 10.1111/gbb.12087 PMC3863782 24034579

[36] Christen V, Mittner F, Fent K. Molecular effects of neonicotinoids in honey bees (Apis mellifera). Environ Sci Technol. 2016;50:4071–81. 10.1021/acs.est.6b00678 26990785

[37] Migdal P, Murawska A, Berbeć E, Plotnik M, Skorus A, Latarowski K. Selected biochemical markers change after oral administration of pesticide mixtures in honey bees. Toxics. 2022;10:590. 10.3390/toxics10100590 PMC9609372 36287870

[38] Watson SA, McStay GP. Functions of cytochrome c oxidase assembly factors. Int J Mol Sci. 2020;19:7254. 10.3390/ijms21197254 PMC7582755 33008142

[39] Fontanesi F, Soto IC, Horn D, Barrientos A. Assembly of mitochondrial cytochrome c-oxidase, a complicated and highly regulated cellular process. Am J Physiol Cell Physiol. 2006;291:C1129–47. 10.1152/ajpcell.00233.2006 16760263

[40] Aggeler R, Capaldi RA. Yeast cytochrome c oxidase subunit VII is essential for assembly of an active enzyme. Cloning, sequencing and characterization of the nuclear-encoded gene. J Biol Chem. 1990;265:16389–93. 2168889

[41] Calder KM, McEwen JE. Deletion of the COX7 gene in Saccharomyces cerevisiae reveals a role for cytochrome c oxidase subunit VII in assembly of remaining subunits. Mol Microbiol. 1991;5:1769–77. 10.1111/j.1365-2958.1991.tb01926.x 1658541

[42] Mouchiroud L, Houtkooper RH, Moullan N, Katsyuba E, Ryu D, Cantó C et al. XThe NAD+/sirtuin pathway modulates longevity through activation of mitochondrial UPR and FOXO signaling. Cell. 2013;154:430–41. 10.1016/j.cell.2013.06.016 PMC3753670 23870130

[43] Houtkooper RH, Mouchiroud L, Ryu D, Moullan N, Katsyuba E, Knott G et al. Mitonuclear protein imbalance as a conserved longevity mechanism. Nature. 2013;497:451–7. 10.1038/nature12188 PMC3663447 23698443

[44] Smirnov A, Entelis N, Martin RP, Tarassov I. Biological significance of 5s rRNA import into human mitochondria: role of ribosomal protein MRP-L18. Genes Dev. 2011;25:1289–1305. 10.1101/gad.624711 PMC3127430 21685364

[45] Greber BJ, Boehringer D, Leibundgut M, Bieri P, Leitner A, Schmitz N et al. The complete structure of the large subunit of the mammalian mitochondrial ribosome. Nature. 2014;515:283–6. 10.1038/nature13895 25271403

[46] Ueno T, Takeuchi H, Kawasaki K, Kubo T. Changes in the gene expression profiles of the hypopharyngeal gland of worker honeybees in association with worker behavior and hormonal factors. PLoS ONE. 2015;10:e0130206. 10.1371/journal.pone.0130206 PMC4470657 26083737

[47] Christen V, Schirrmann M, Frey JE, Fent K. Global Transcriptomic effects of environmentally relevant concentrations of the neonicotinoids Clothianidin, Imidacloprid, and Thiamethoxam in the brain of Honey bees (Apis mellifera). Environ Sci Technol. 2018;52:7534–44. 10.1021/acs.est.8b01801 29851480

[48] Ohashi K, Natori S, Kubo T. Change in the mode of gene expression of the hypopharyngeal gland cells with an age-dependent role change of the worker honeybee Apis mellifera L. Eur J Biochem. 1997;249:797–802. 10.1111/j.1432-1033.1997.t01-1-00797.x 9395329

[49] McKinlay A. Radiotelephones and human health: a European research initiative. Radiat Prot Dosimetry. 1997;72:313–20.

[50] Wagner JDO, Jankowsky E, Company M, Pyle AM, Abelson JN. The DEAH-box protein PRP22 is an ATPase that mediates ATP-dependent mRNA release from the spliceosome and unwinds RNA duplexes. EMBO J. 1998;17:2926–37. 10.1093/emboj/17.10.2926 PMC1170633 9582286

[51] Mann K, Röschke J. Effects of pulsed high-frequency electromagnetic fields on human sleep. Neuropsychobiology. 1996;33:41–7. 10.1159/000119247 8821374

[52] Edelstyn N, Oldershaw A. The acute effects of exposure to the electromagnetic field emitted by mobile phones on human attention. NeuroReport. 2002;13:119–21. 10.1097/00001756-200201210-00028 11924872

[53] Haarala C, Björnberg L, Ek M, Laine M, Revonsuo A, Koivisto M et al. Effect of a 902 MHz electromagnetic field emitted by mobile phones on human cognitive function: a replication study. Bioelectromagnetics. 2003;24:e43478. 10.1002/bem.10105 12696088

[54] Junkersdorf B, Bauer H, Gutzeit HO. Electromagnetic fields enhance the stress response at elevated temperatures in the nematode Caenorhabditis elegans. Bioelectromagnetics. 2000;21:100–6. 10.1002/(sici)1521-186x(200002)21:2<100::aid-bem4>3.0.co;2-u 10653620

[55] Khalil AM, Qassem W (1991). Cytogenetic effects of pulsing electromagnetic field of human lymphocytes in vitro: chromosome aberrations, sister-chromatid exchanges and cell kinetics. Mutat Research/Fundamental Mol Mech Mutagen..

[56] Liu Y, Liu W, Bin, Liu KJ, Ao L, Zhong JL, Cao J, et al. Effect of 50 Hz Extremely low-frequency electromagnetic fields on the DNA methylation and DNA methyltransferases in mouse spermatocyte-derived cell line GC-2. Biomed Res Int. 2015;2015:237183. 10.1155/2015/237183 PMC4538330 26339596

[57] Sakhnini L, Al-Ghareeb S, Khalil S, Ahmed R, Abdul Ameer A, Kamal A. Effects of exposure to 50Hz electromagnetic fields on Morris water-maze performance of prenatal and neonatal mice. J Association Arab Universities Basic Appl Sci. 2014;15:1–5.

[58] Foroozandeh E, Derakhshan-Barjoei P, Jadidi M. Toxic effects of 50 hz electromagnetic field on memory consolidation in male and female mice. Toxicol Ind Health. 2013;29:293–9. 10.1177/0748233711433931 22397835

[59] Luo X, Jia S, Li R, Gao P, Zhang Y. Occupational exposure to 50 hz magnetic fields does not alter responses of inflammatory genes and activation of splenic lymphocytes in mice. Int J Occup Med Environ Health. 2016;29:277–91 10.13075/ijomeh.1896.00519 26670356

[60] Kullnick U, Lüthe LC, Wolff HG. Do weak, low pulsed frequency, high-frequency electromagnetic or magnetic fields alter the basic bioelectrical parameters of nerve cells in vineyard snails (Helix pomatia L.)? II. Magnetic fields. Bioelectrochem Bioenerg. 1995;37:39–45.

[61] Ossenkopp K-P, Kavaliers M, Lipa S. Increased mortality in land snails (Cepaea nemoralis) exposed to powerline (60-Hz) magnetic fields and effects of the light-dark cycle. Neurosci Lett [Internet]. 1990;114:89–94. https://www.sciencedirect.com/science/article/pii/030439409090433A. 10.1016/0304-3940(90)90433-a 2381576

