# Supplementary material for: Metabolism gene expression in worker honey bees after exposure to 50Hz electric field - semi-field analysis
Source: Front Zool. 2024 May 29;21:14. doi: 10.1186/s12983-024-00535-1 (PMC11134740; doi:10.1186/s12983-024-00535-1)
Supplement: Supplementary file 1 — Supplementary Material 1. [file 12983_2024_535_MOESM1_ESM.docx]

Wrocław, 17^th^ May 2024

**EXPLANATION LETTER**

Dear Editors,

Subject: Submission of the reviewed paper untitled “Metabolism gene expression in worker honey bees after exposure to 50Hz electric field frequency - semi-field analysis**’’** no. FINZ-D-24-00024.

Thank you for your email dated 9.05.2024 enclosing the reviewers’ comments. Our responses are given in a point-by-point manner below and in the manuscript track changes.

Sincerely,
Agnieszka Murawska

Reviewer reports:

Reviewer #3: 1.E-field effects and EMF effects have been discussed in a way lack of logical in the discussion part. I suggest authors to organize the discussion part better, e.g., discuss the electric effects, magnetic effects or the effects triggered by both electric and magnetic field, separately or logically.

*We changed order of paragraphs, separated effects of radio and extremely low frequencies and we hope that now it is more clear*.

2. Double check the typos. E.g., no full name for EMF in the manuscript.

*We checked typos and errors throughout the manuscript.*
